# Predictive value of preoperative T1 slope minus cervical lordosis for clinical outcomes after standalone laminectomy in elderly degenerative cervical myelopathy

**DOI:** 10.1038/s41598-026-46868-1

**Published:** 2026-04-13

**Authors:** Ahmed Mohammed Ragab, Mahmoud M. Taha, Mansour Abdel Mageed Makkia, Ayman M. Ismail

**Affiliations:** 1 Department of Neurosurgery, Faculty of Medicine, Zagazig University, Zagazig, Sharkia Egypt; 2 Department of Neurosurgery, Faculty of Medicine, Kafrelsheikh University, Kafrelsheikh, Egypt

**Keywords:** Degenerative cervical myelopathy, Laminectomy, Sagittal cervical alignment, Lordosis, T1 slope minus lordosis, Predictive modeling, Diseases, Medical research, Neurology, Risk factors

## Abstract

Standalone cervical laminectomy is frequently performed in elderly patients with degenerative cervical myelopathy (DCM), yet practical prognostic determinants remain limited. We examined whether the preoperative T1 slope minus cervical lordosis (T1S-CL) predicts 2-year clinical outcomes and yields clinically useful decision thresholds. In this retrospective cohort of elderly DCM patients who underwent multilevel standalone laminectomy with a minimum 2-year follow-up, outcomes were assessed using the modified Japanese Orthopaedic Association (mJOA) score and Visual Analog Scale (VAS) for neck and arm pain. The primary endpoint was achievement of the minimal clinically important difference (MCID), prespecified as a ≥ 2-point increase in mJOA. Cervical alignment parameters including T1 slope (T1S), C2–C7 lordosis (CL), and T1S-CL were measured pre- and postoperatively. Predictive performance was evaluated using receiver operating characteristic (ROC) analysis and multivariable logistic regression with internal bootstrap validation. Among 68 patients (mean age 68.38 ± 3.15 years), 82.4% achieved MCID at 2 years. Laminectomy was associated with a modest decline in lordosis, and a corresponding increase in T1S-CL. Preoperative T1S-CL independently predicted MCID achievement (adjusted odds ratio per 1° = 0.556; *p* < 0.001) and demonstrated superior discrimination compared with T1S or CL alone. A two-threshold strategy emerged: T1S-CL ≤ 16.5° showed high specificity (91.7%) and positive predictive value (97.2%) for favourable outcome, whereas T1S-CL > 20° provided strong sensitivity (94.6%) for identifying poor outcome. These findings support T1S-CL as a promising preoperative predictor for exploratory risk stratification. However, the proposed thresholds require external validation before routine clinical use.

**Supplementary Information:**

The online version contains supplementary material available at 10.1038/s41598-026-46868-1.

## Introduction

Degenerative cervical myelopathy (DCM) is the most common progressive, non-traumatic spinal cord disorder in adults, and its prevalence increases in parallel with rising life expectancy^1^. Over time, degenerative changes progressively narrow the spinal canal, resulting in neuronal ischemia and chronic cord compression^2^. Clinically, DCM presents as a spectrum of motor and sensory deficits, including gait imbalance, hand clumsiness, impaired coordination, paraesthesia, axial neck pain, and sphincter dysfunction^3,4^. If left untreated, DCM may lead to significant disability and poor quality of life^5^.

The initial management of mild DCM often involves nonoperative strategies and careful monitoring. However, the natural history of moderate to severe myelopathy frequently involves progressive deterioration in up to 60% of patients over 5 years^6^. Surgical decompression is therefore considered superior in moderate to severe cases, as it halts neurologic decline and facilitates functional recovery^7^. The optimal approach remains under debate and may vary depending on the number of levels involved, location of pathology, baseline cervical sagittal alignment, and patient comorbidities^8,9^. Posterior cervical laminectomy without instrumented fusion offers several advantages including motion preservation, reduced surgical morbidity, and avoidance of hardware-related complications. However, concerns persist regarding postoperative kyphotic progression due to impairment of posterior cervical muscle-ligament complex^10^.

Preoperative cervical sagittal parameters are increasingly being implicated in postoperative alignment and patient reported outcomes across cervical procedures. Previous studies in laminoplasty cohorts reported that a higher T1 slope (T1S) was associated with greater loss of C2–C7 lordosis (CL) and kyphotic shift at follow-up^11,12^, while other studies reported no independent effect of T1S after adjustment^13,14^. Building on the mechanistic link between T1S (lordotic demand) and C2–7 lordosis (capacity), T1 slope minus cervical lordosis (T1S-CL) has been proposed as the cervical analog of pelvic incidence minus lumbar lordosis (PI–LL), with growing evidence that larger mismatch tracks worse health-related quality of life, particularly in posterior reconstruction^15,16^ and laminoplasty^17–19^. However, meta-analytic synthesis indicates that correlations between T1S-CL and patient-reported outcomes are modest on average, underscoring the need for adjusted models and internal validation^20^. Despite this body of work in laminoplasty and reconstruction cohorts, the predictive role of preoperative T1S-CL for clinical outcomes in elderly patients undergoing standalone laminectomy remains unclear, leaving a clinically relevant evidence gap.

To evaluate the relationship between preoperative T1slope minus cervical lordosis and the 2-year clinical outcomes of standalone laminectomy in elderly patients with DCM, this study had prespecified 4 aims: (1) quantify the 2-year clinical and radiological changes after standalone laminectomy; (2) compare these changes between patients who did and did not achieve the minimal clinically important difference (MCID); (3) assess the predictive performance of preoperative T1S-CL, against T1S and CL, using Receiver Operating Characteristic (ROC) analysis and identify clinically usable cut-off points; and (4) internally validate a parsimonious multivariable model integrating T1S-CL with key covariates, reporting discrimination, calibration, and decision-oriented thresholds.

## Methods

### Study design

This retrospective observational cohort study included 68 consecutive elderly patients who underwent posterior cervical laminectomy without fusion for degenerative cervical myelopathy in Neurosurgery Department, Faculty of Medicine, Zagazig University, Egypt, from January 2019 to January 2023. The study protocol was approved by the Zagazig University Faculty of Medicine Institutional Review Board (ZU-IRB), Zagazig, Egypt (Approval No. ZU-IRB #197/10-3-2024). Written informed consent for surgery was obtained from all patients prior to their procedures. For this retrospective review of medical records, the requirement for informed consent to participate in research was waived by the Zagazig University Faculty of Medicine Institutional Review Board owing to the use of de-identified data and minimal risk to participants. All methods were performed in accordance with the Declaration of Helsinki and relevant institutional and national guidelines and regulations, and reporting adhered to STROBE recommendations.

### Eligibility criteria

Patients older than 65 years, of either sex, who presented with symptomatic degenerative cervical myelopathy, with magnetic resonance imaging (MRI)-confirmed stenosis and multilevel spinal cord compression, who underwent multilevel posterior cervical laminectomy without fusion, and completed a minimum of 2-year postoperative follow-up were included. Patients were excluded if they had previous cervical spine surgery, myelopathy secondary to trauma, neoplasm, ossification of the posterior longitudinal ligament (OPLL) in preoperative C.T (computed tomography), Chiari malformation, or inadequate imaging. Patients with preoperative kyphosis, C2–C7 lordosis < 5°, or sagittal plane instability (defined as > 3.5 mm translation or > 11° angulation on preoperative dynamic radiographs) were also excluded.

### Surgical approach

Surgery was performed by certified neurosurgeons using standard anesthetic and operative techniques. Fiber-optic intubation was used for difficult airways or limited neck extension. Intraoperative hypotension was avoided to maintain adequate spinal cord perfusion. Patients were positioned prone with eye protection, slight cervical flexion, and reverse Trendelenburg to reduce venous pooling. through a classic midline approach, the spinous processes were approached through the midline raphe to minimize muscle bleeding and postoperative neck pain. A strict subperiosteal plane was then maintained with utmost care taken to preserve nuchal attachments to the C2 spinous process. The facet joints were carefully preserved during laminectomy.

### Data collection

#### clinical data

From the medical records, the following demographic and clinical parameters were systematically extracted for each patient: age, sex, body mass index (BMI), current smoking status, comorbidities, duration of symptoms, preoperative Visual Analog Scale (VAS) for neck and arm pain, preoperative modified Japanese Orthopaedic Association (mJOA) score, surgery duration, estimated blood loss, number of operated levels, postoperative complications, follow up duration, and 2-year postoperative VAS scores for neck and arm pain, mJOA score. All data were double-checked for accuracy and completeness by two independent reviewers.

#### Radiological parameters and measurements

**Fig. 1:**
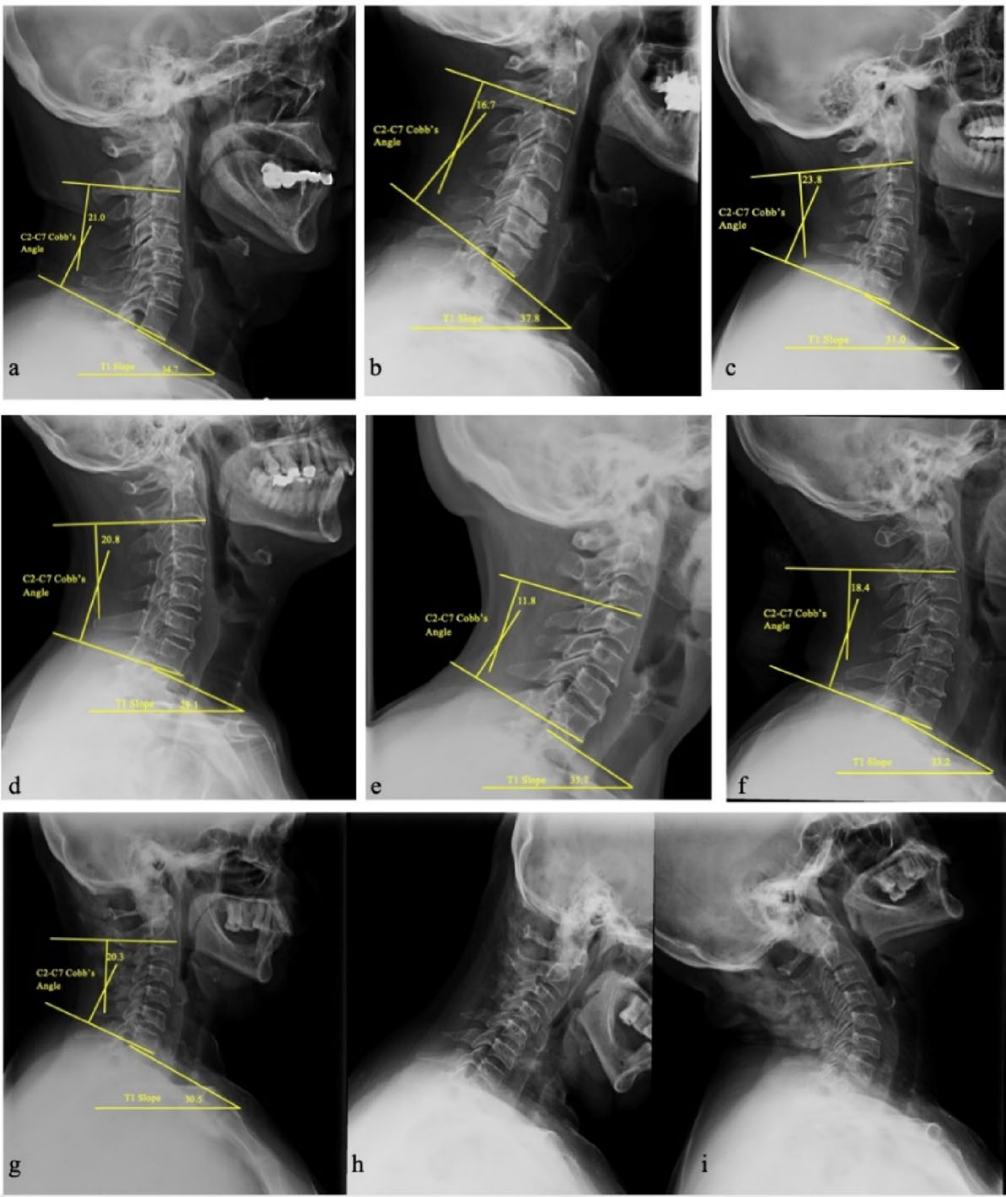
Preoperative lateral cervical X-ray with measurements of cervical alignment. Neutral standing lateral views **(a–g)** illustrate measurement of C2–C7 lordosis as the Cobb angle between the inferior endplates of C2 and C7, and T1 slope as the angle between the T1 superior endplate and the horizontal plane. Dynamic flexion–extension views **(h–i)** demonstrate absence of translational (> 3.5 mm) or angular (> 11°) sagittal instability.

**Fig. 2:**
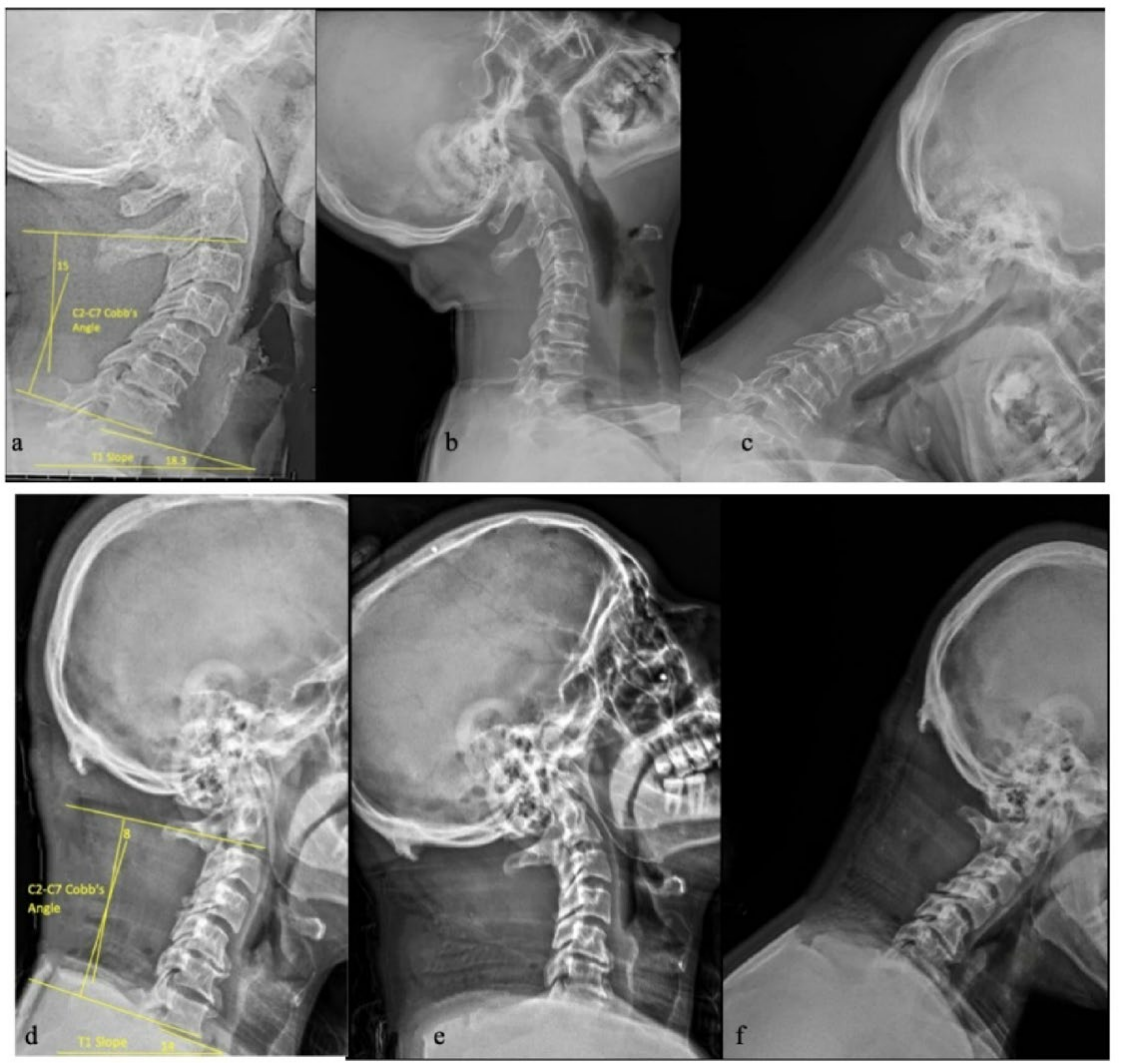
Postoperative lateral cervical spine X-ray at 2-year postoperative follow-up. Neutral standing lateral views **(a, d)** and flexion–extension views **(b, c, e, f)** following multilevel laminectomy show preserved motion without postoperative sagittal instability. C2–C7 lordosis and T1 slope were re-measured using the same method as baseline.

Standing lateral radiographs obtained within 3 months before surgery and within a ± 3‑month window around the 24‑month visit were used for the measurements. All standing lateral cervical radiographs were obtained according to the same institutional protocol, with patients in a neutral standing position maintaining natural horizontal gaze, and with the upper limbs relaxed at the sides. Two raters (radiologist and spine surgeon), blinded to the outcomes and not involved in the study, measured C2–C7 lordosis (the angle between the inferior plate of C2 and the inferior end plate of C7) and T1 slope (the angle between the T1 superior endplate and the horizontal line). The (T1S-CL) was computed as T1 slope minus C2–C7 lordosis. Disagreements > 2° were resolved by consensus and the mean of paired readings was analyzed. Inter-rater reliability was assessed using the intraclass correlation coefficient (ICC), applying a two-way random effects model with absolute agreement. ICC values were interpreted as poor (< 0.5), moderate (0.5–0.75), good (0.75–0.9), or excellent (> 0.9), following established guidelines^21^. Dynamic lateral flexion/extension plain radiographs were assessed for sagittal plane instability (> 3.5 mm translation or > 11° angulation) (Figs. 1 and 2).

**Fig. 3:**
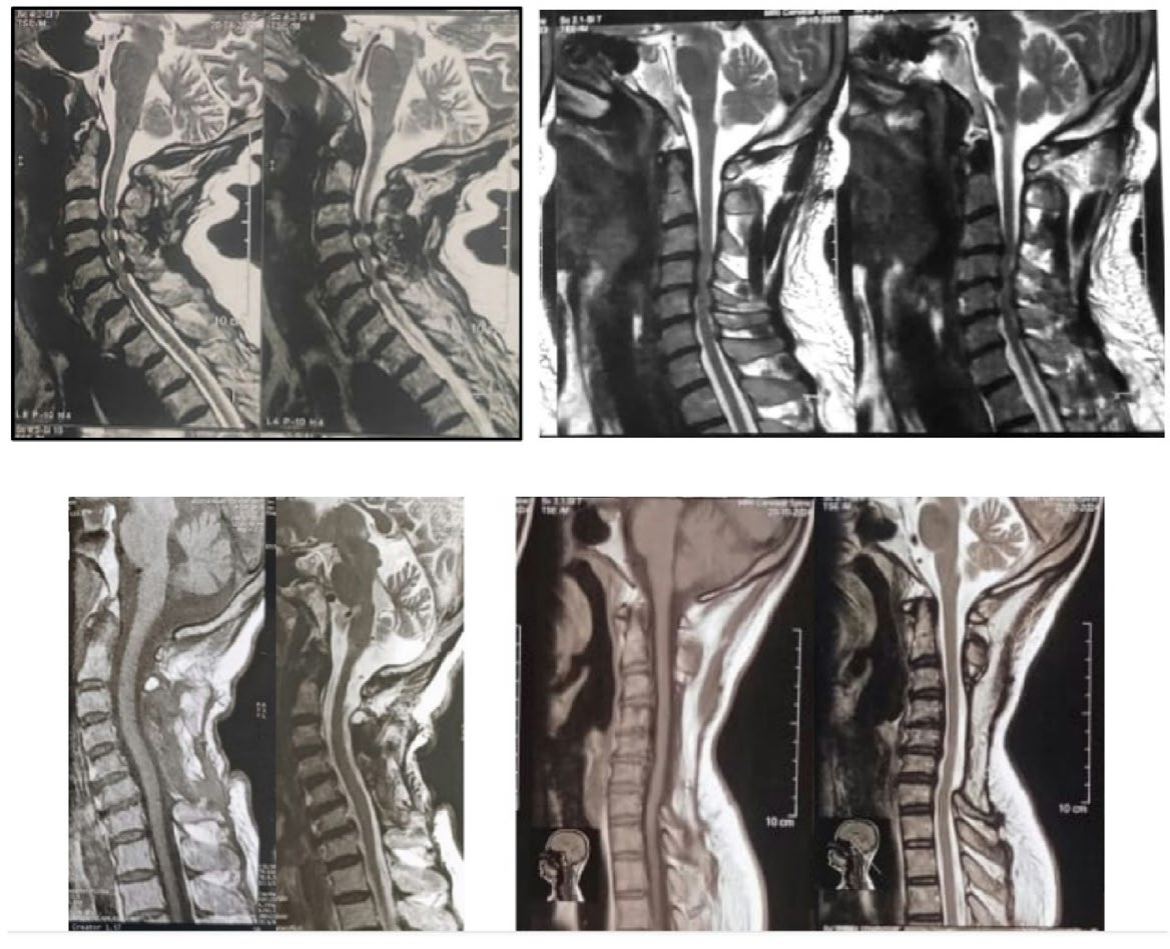
Preoperative and follow-up MRI. Preoperative (upper row) and follow-up (lower row) MRI images. Multi-level spinal cord compressions with T2-hyperintense cord signal noted in the preoperative images. Postoperative follow-up images showing adequate spinal cord decompression, and modest kyphotic progression after multilevel laminectomy.

Preoperative MRI was examined for levels of maximum compression, degree of compression, multilevel stenosis, spinal cord T2 hyperintensity, disc-osteophyte complexes, ligamental hypertrophy and facet arthropathy. 2-year postoperative MRI images were examined for adequacy of spinal cord decompression, residual canal compromise, and spinal cord T2 hyperintensity signals (Fig. 3).

### Outcome measures

The primary endpoint was achievement of the minimal clinically important difference (MCID) at 2 years prespecified as ≥ 2 points improvement in the mJOA score which is consistent with prior literature^22,23^. Secondary outcome measures were perioperative complications and within patient changes from baseline to 2 years in VAS-Neck, VAS-Arm, mJOA, C2–C7 lordosis, T1 slope, and T1S-CL.

### Sample size consideration

For univariable screening of radiographic predictors, adequacy was evaluated against an Area Under the Curve (AUC) precision target of ± 0.07 with an expected AUC of 0.80, yielding a minimum of 60 patients for 80% power at α = 0.05.

### Statistical analysis

Statistical analyses were performed using IBM SPSS Statistics for Mac, version 31.0.1.0 (IBM Corp., NY, USA; https://www.ibm.com/products/spss-statistics). Continuous variables were summarized as mean ± SD or median (IQR), as appropriate, and categorical variables as counts (%). Normality was assessed using the Shapiro–Wilk test. Within-group changes were analyzed using the Wilcoxon signed-rank test, and between-group comparisons were performed using the Mann–Whitney U test or chi-square (χ²) test, as appropriate. Analysis of covariance (ANCOVA) was used to compare 2-year outcomes between MCID strata while adjusting for baseline values. Discrimination of preoperative radiographic parameters was assessed using receiver operating characteristic (ROC) analysis with area under the curve (AUC) and 95% confidence intervals (CI), and optimal cutoffs were identified using Youden’s index. For clinical interpretability, threshold performance was additionally summarized by sensitivity, specificity, positive predictive value, negative predictive value, and likelihood ratios. For prognostic modeling, candidate models were compared at the apparent-performance level, including preoperative T1S–CL alone and the final adjusted model incorporating T1S–CL and symptom duration. Because only 12 patients did not achieve MCID at 2 years, the number of outcome events available for multivariable regression was limited; accordingly, the final adjusted model was intentionally restricted to 2 predictors, yielding an events-per-variable ratio of approximately 6, and the findings were interpreted as exploratory rather than definitive. Logistic regression was used with MCID achievement as the dependent variable and T1S–CL as the primary predictor of interest. Internal validation was performed using nonparametric bootstrapping with 2,000 resamples to assess coefficient stability and calibration-related measures. Model performance was summarized using apparent AUC (95% CI), calibration slope and intercept from a logistic calibration model, Nagelkerke R², Hosmer–Lemeshow goodness-of-fit test, and Brier score. All tests were two-sided, and *p* < 0.05 was considered statistically significant.

## Results

### study flow

**Fig. 4:**
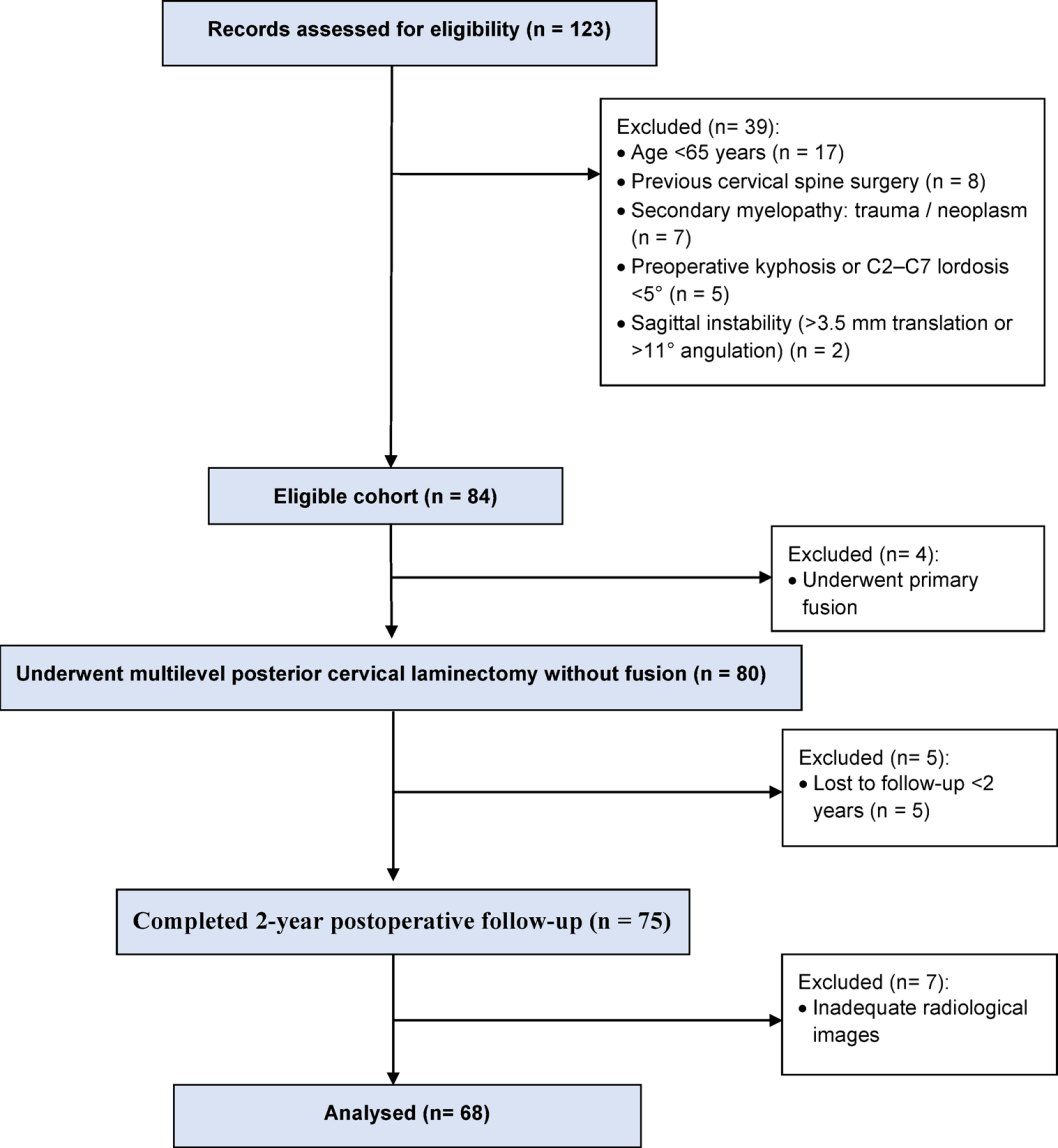
Study flow diagram. Screening, eligibility, exclusions and final analytic cohort (*n* = 68) with reasons for exclusion and follow-up completeness at 2-year.

As demonstrated in Fig. 4,55 of the 123 reviewed patients were excluded, leaving 68 patients with no missing data in the final cohort who were included in the final analysis.

### Patients characteristics

**Fig. 5:**
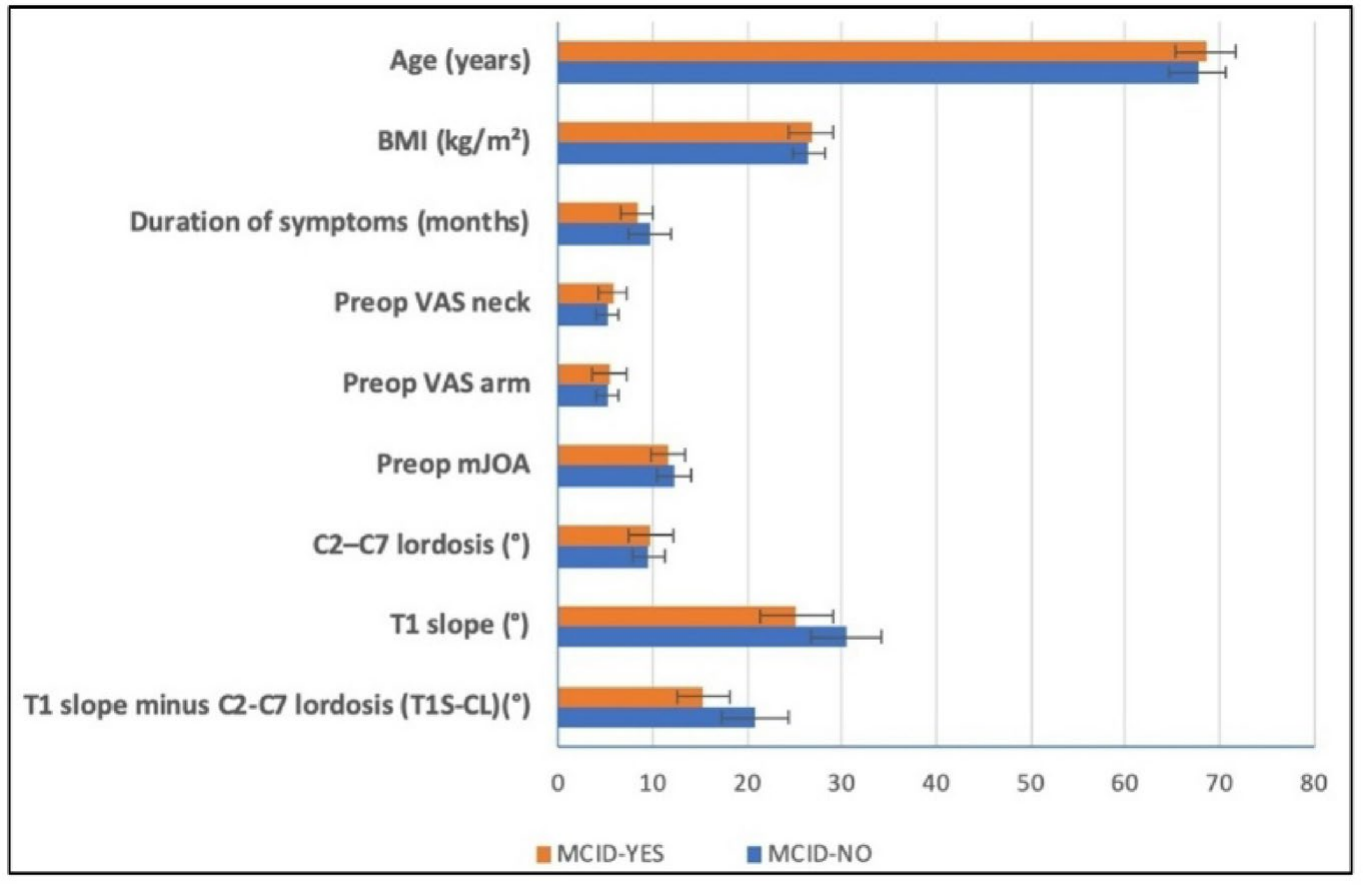
Baseline patients’ characteristics stratified by MCID achievement (continuous variables). Distributions of age, BMI, symptom duration, VAS-neck, VAS-arm, mJOA, C2-C7 lordosis, T1 Slope, and T1Slope minus cervical lordosis at baseline, stratified by MCID achievement at 2-year postoperative. Error bars indicate standard deviations. Abbreviations: BMI, body mass index; VAS, visual analogue scale; mJOA, modified Japanese Orthopaedic Association; MCID, minimal clinically important difference.

**Fig. 6:**
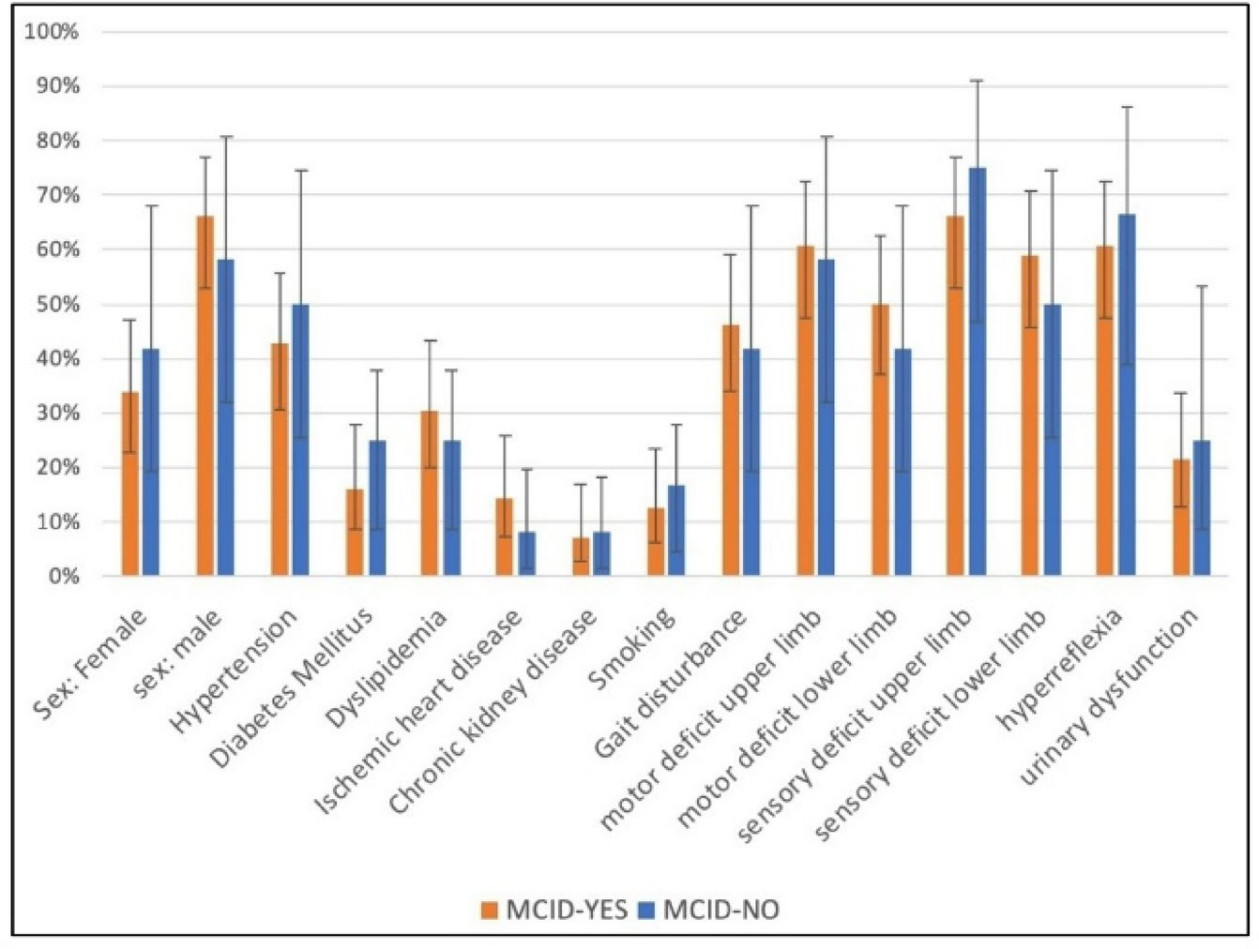
Baseline patients’ characteristics stratified by MCID achievement (categorical variables). Proportions for sex, smoking status, comorbidities (diabetes mellitus, hypertension, ischemic heart disease, dyslipidemia, chronic kidney disease), neurological signs (gait disturbance, upper/lower limb motor/sensory deficit, hyperreflexia, urinary dysfunction) in MCID-Yes vs. MCID-No groups. Error bars indicate 95% CIs. Abbreviations: MCID, minimal clinically important difference.

Table 1, and Figs. 5 and 6 show the baseline patients’ characteristics. This study comprised 68 patients with mean age of 68.4 ± 3.2 years, 64.7% male and 35.3% female (male to female ratio, 1.8:1). Baseline characteristics were broadly similar between MCID-Yes (*n* = 56) and MCID-No (*n* = 12) groups for age, sex, BMI, comorbidities, neurological status, and C2–C7 lordosis. T1 slope was lower in MCID-Yes group in comparison to MCID-No group (25.2 ± 3.9° vs. 30.5 ± 3.7°, *p* < 0.001), as was T1 slope minus C2-C7 lordosis (T1S-CL) (15.4 ± 2.8° vs. 20.9 ± 3.6°, *p* < 0.001). In addition, MCID-Yes patients had a shorter symptom duration at baseline (8.4 ± 1.7 vs. 9.7 ± 2.2 months, *p* = 0.028).

**Table 1:** Baseline characteristics, overall and by MCID achievement at 2-year.

Age (years)	Overall (*n* = 68)	MCID-Yes (*n* = 56)	MCID-No (*n* = 12)	*P*-value
	68.38 ± 3.15	68.54 ± 3.19	67.67 ± 2.99	0.390^a^
Sex:				χ²
Male	44 (64.7%)	37(66.1%)	7 (58.3%)	0.611
female	24(35.3%)	19 (33.9%)	5 (41.7%)	
Body Mass Index (BMI) (kg/m²)	26.71 ± 2.30	26.75 ± 2.41	26.50 ± 1.78	0.735^a^
Comorbidities:				χ²
Diabetes Mellitus	12 (17.6%)	9 (16.1%)	3 (25%)	0.462
Hypertension	30 (44.1%)	24 (42.9%)	6 (50%)	0.651
Dyslipidemia	20 (29.4%)	17 (30.4%)	3 (25%)	0.712
Ischemic Heart Disease	9 (13.2%)	8 (14.3%)	1(8.3%)	0.581
Chronic Kidney Disease	5 (7.4%)	4(7.1%)	1(8.3%)	0.886
Smoking	9 (13.2%)	7 (12.5%)	2 (16.7%)	0.699
Duration of symptoms (months)	8.60 ± 1.86	8.37 ± 1.71	9.67 ± 2.23	0.028^a*^
Preop. VAS neck	5.69 ± 1.47	5.8 ± 1.52	5.17 ± 1.12	0.150^b^
Preop. VAS arm	5.38 ± 1.66	5.41 ± 1.76	5.25 ± 1.14	0.768^b^
Preop. mJOA	11.75 ± 1.86	11.64 ± 1.87	12.25 ± 1.82	0.275^b^
Neurological status, preop				χ²
Gait disturbance	31(45.6%)	26(46.4%)	5(41.7%)	0.764
Motor deficit in upper limbs	41(60.3%)	34(60.7%)	7(58.3%)	0.878
Motor deficit in lower limbs	33(48.5%)	28(50%)	5(41.7%)	0.600
Sensory deficit in upper limbs	46(67.6%)	37(66.1%)	9(75%)	0.549
Sensory deficit in lower limbs	39(57.4%)	33(58.9%)	6(50%)	0.570
Hyperreflexia	42(61.8%)	34(60.7%)	8(66.7%)	0.700
Urinary dysfunction	15(22.1%)	12(21.4%)	3(25%)	0.787
Preop. C2–C7 lordosis (°)	9.76 ± 2.2	9.8 ± 2.3	9.58 ± 1.73	0.620^b^
Preop. T1 slope (°)	26.1 ± 4.34	25.16 ± 3.88	30.5 ± 3.73	<.001^a^**
Preop. T1 slope minus C2-C7 lordosis (T1S-CL) (°)	16.34 ± 3.63	15.36 ± 2.82	20.92 ± 3.55	<.001^b^**

a: Independent sample t-test, b: Mann-Whitney U test, χ²: Chi-Square, * *p* < 0.05: statistically significant, **p < 0.001: statistically highly significant, VAS: Visual Analog Scale, mJOA: modified Japanese Orthopaedic Association (mJOA) score, (°): degrees, MCID: Minimal Clinically Important Difference, preop.: preoperative.

### Operative characteristics

As shown in Table 2, the operative time, intraoperative blood loss and number of operated levels were comparable between MCID-Yes and in MCID-No groups with no statistically significant differences. Mean postoperative follow-up duration was 29.2 ± 2.6 months overall. Five patients (7.4%) developed surgical site infection, with two patients requiring surgical debridement, three patients (4.4%) developed C5 palsy, four patients (5.9%) had postoperative chest infection and one patient (1.5%) developed DVT. Perioperative complications were comparable between the two groups.

**Table 2:** Operative characteristics, perioperative complications and follow-up duration overall and by MCID achievement at 2-year.

Operative time, minutes (mean ± SD)	Overall (*n*−68)	MCID-Yes (*n* = 56)	MCID-No (*n* = 12)	*p*-value
	133.47 ± 23.67	134.82 ± 24.14	127.17 ± 21.11	0.313^a^
**Intraoperative blood loss (ml)** (mean ± SD)	194.24 ± 42.18	193.21 ± 39.93	199 ± 53.27	0.670 ^a^
**Postoperative follow-up duration**,** months** (mean ± SD)	29.19 ± 2.55	29.02 ± 2.32	30 ± 3.46	0.229 ^a^
**Number of operated levels**, n (%)				χ²
− 3 levels	24 (35.3%)	20(35.7%)	4 (33.3%)	0.213
− 4 levels	40 (58.8%)	34 (60.7%)	6 (50%)	
− 5 levels	4 (5.9%)	2 (3.6%)	2 (16.7%)	
**Perioperative complications**, n (%)				χ²
-Surgical site infection	5 (7.4%)	3 (5.4%)	2 (16.7%)	0.173
-C5 palsy	3 (4.4%)	2 (3.6%)	1(8.3%)	0.466
-Chest infection	4 (5.9%)	3 (5.4%)	1(8.3%)	0.691
-DVT	1 (1.5%)	1(1.8%)	0 (0%)	0.641

a: Independent sample t-test, χ²: chi-square, DVT: Deep venous Thrombosis, MCID: Minimal Clinically Important Difference.

### Clinical outcomes

As shown in Table 3, at 2-year postoperative, the patients showed large clinically meaningful improvements across all outcomes. VAS-neck declined from 5.69 ± 1.47 to 2.66 ± 1.25 (change − 3.03, 95% CI − 3.47 to − 2.59, *p* < 0.001). VAS-arm declined from 5.38 ± 1.66 to 2.84 ± 1.13 (change − 2.54, 95% CI − 3.06 to −2.02, *p* < 0 0.001). mJOA improved from 11.75 ± 1.86 to 13.82 ± 1.88 (change + 2.07, 95% CI 1.81 to 2.34, *p* < 0.001).

Stratification of patients based on achievement of the minimal clinically important difference (MCID) at 2 years defined as ≥ 2 points improvement in mJOA (Table 4) showed that the magnitude of change favored the MCID-Yes group. For VAS-neck, the between-group difference in change was 2.26 (95% CI 1.24–3.29), and ANCOVA adjusted for baseline VAS-neck indicated a significant group effect (*p* < 0.001). For VAS-arm, the between-group difference in change was (0.76, 95% CI − 0.36 to 1.89), however, ANCOVA adjusted for baseline VAS-arm indicated no significant group effect (*p* = 0.123). For mJOA, the between-group difference in change was 2.21 (95% CI 1.71–2.72), and ANCOVA adjusted for baseline mJOA confirmed a significant group effect (*p* < 0.001).

**Table 3:** Overall clinical outcomes at 2-year.

Outcome	Preoperative (mean ± SD)	2- years Postoperative (mean ± SD)	Change (2years postoperative -Preoperative)			*p*-value
			mean	95% CI		
				lower	upper	
**VAS-Neck**	5.69 ± 1.47	2.66 ± 1.25	− 3.03	− 3.47	− 2.59	<.001^a^**
**VAS-Arm**	5.38 ± 1.66	2.84 ± 1.13	− 2.54	− 3.06	−2.02	<.001^a^**
**mJOA**	11.75 ± 1.86	13.82 ± 1.88	2.07	1.81	2.34	<.001^a^**

a: Wilcoxon Signed Rank Test, VAS: Visual Analog Scale, mJOA: modified Japanese Orthopaedic Association (mJOA) score, CI: Confidence Interval, **p < 0.001: statistically highly significant.

**Table 4:** Clinical outcomes by MCID achievement at 2-year with baseline-adjusted comparisons.

**Outcome**	**Group**	**Preoperative (mean ± SD)**	**2-years Postoperative (mean ± SD)**	**Change (2years postoperative -Preoperative)**			**Within-group** **difference** **(p)**	**Between group** **difference** **in change** **(Mean, 95% CI)**	**Between group** **Difference** **(P)**	**Adjusted Between-group difference** **(ANCOVA)** **(p)**
				**Mean**	**95% CI**					
					**lower**	**upper**				
**VAS-Neck**	**MCID- Yes (n=56)**	5.8±1.52	2.38 ± 1.11	-3.43	-3.90	-2.96	<.001^a^**	2.26 (1.24-3.29)	<0.001^b^**	<0.001^c^**
	**MCID- No (n=12)**	5.17±1.12	4.00 ± 1.04	-1.17	-1.53	-0.80	0.002^a^*			
**VAS-Arm**	**MCID- Yes**	5.41±1.76	2.73± 1.14	-2.68	-3.28	-2.08	<.001^a^**	0.76(-0.36-1.89)	0.295^b^	0.123^c^
	**MCID- No**	5.25 ± 1.14	3.33 ± 0.99	- 1.92	- 2.91	-0.92	0.011^a^*			
**mJOA**	**MCID -Yes**	11.64±1.87	14.11 ± 1.83	2.46	2.28	2.65	<.001^a^**	2.21 (1.71-2.72)	0.007^b^*	<0.001^c^**
	**MCID -No**	12.25±1.82	12.50± 1.62	0.25	-0.23	0.73	0.257^a^			

a: Wilcoxon Signed Rank Test, b: Mann-Whitney U test, c: Analysis of Covariance (ANCOVA), * p< 0.05: statistically significant, **p< 0.001: statistically highly significant, T1S-CL: T1slope minus C2-C7 lordosis, MCID: Minimal Clinically Important Difference.

### Radiological outcomes

As shown in Table 5, at 2-year postoperative, the patients demonstrated statistically significant changes in cervical alignment. C2–C7 lordosis decreased from 9.76 ± 2.20° preoperatively to 5.90 ± 2.62° at 2-years postoperative (*p* < 0.001). In addition, T1 slope showed significant reduction from 26.10 ± 4.34° preoperatively to 23.22 ± 3.97° at 2-years (*p* < 0.001). Because the loss of lordosis was proportionally greater than the reduction in T1 slope, the derived T1S-CL increased from 16.34 ± 3.63° preoperatively to 17.32 ± 3.85° at 2-years postoperative (*p* = 0.012).

Table 6 shows the radiological outcomes stratified by MCID achievement at 2-year. Over 2 years, both groups lost C2–C7 lordosis, but this decline did not differ by MCID status (between-group difference 1.27°, *p* = 0.271, ANCOVA adjusted for base line C2-C7 lordosis *p* = 0.669), indicating that lordosis alone does not explain clinical improvement. In contrast, sagittal balance metrics were discriminative: although raw change in T1 slope did not differ (change − 0.87°; *p* = 0.594), baseline-adjusted analysis showed a significant group effect (ANCOVA *p* = 0.003), with higher sustained T1 slope among MCID-No group. Most importantly, the T1S-CL remained stable in MCID-Yes (mean + 0.61°, *p* = 0.114) but worsened in non-achievers (+ 2.75°, *p* = 0.018), yielding a significant between-group difference (change − 2.14°, *p* = 0.039) that persisted after adjustment (ANCOVA *p* < 0.001). Taken together, postoperative sagittal mismatch (T1S-CL), not absolute lordosis, best tracked clinically meaningful recovery.

The inter-rater agreement for measurement of radiological parameters, as shown in Table 7, was excellent for all measured parameters. The intraclass correlation coefficient (ICC) for T1 slope, cervical lordosis, and TS–CL ranged from 0.943 to 0.985 (*p* < 0.001), indicating strong reliability of measurement.

**Table 5:** Overall radiological outcomes at 2-year.

Outcome	Preoperative (mean ± SD)	2-years Postoperative (mean ± SD)	Change (2years postoperative -Preoperative)			*p*-value
			Mean	95% CI		
				lower	upper	
**C2–C7 lordosis**	9.76 ± 2.2	5.9 ± 2.62	− 3.87	− 4.36	−3.38	<.001^a^**
**T1 slope**	26.1 ± 4.34	23.22 ± 3.97	− 2.88	− 3.80	− 1.96	< 0.001 ^a^**
**T1S-CL**	16.34 ± 3.63	17.32 ± 3.85	0.99	0.29	1.87	0.012 ^a^*

a: Wilcoxon Signed Rank Test, CI: Confidence Interval, * *p* < 0.05: statistically significant, **p < 0.001: statistically highly significant, T1S-CL: T1slope minus C2-C7 lordosis.

**Table 6:** Radiological outcomes by MCID achievement at 2-year with baseline-adjusted comparisons.

Outcome	Group	Preoperative (mean ± SD)	2-years postoperative (mean ± SD)	Change (2years postoperative -Preoperative)			Within-group difference (P)	Between group difference in change (Mean, 95% CI)	Between group difference (p)	Adjusted Between-group difference (ANCOVA) (p)
				Mean	95% CI					
					lower	Upper				
**C2–C7 lordosis**	MCID- Yes (*n* = 56)	9.8 ± 2.3	6.16 ± 2.56	−3.64	−4.13	−3.16	<.001^a^**	1.27(−0.45 to 3.00)	0.271 ^b^	0.669 ^c^
	MCID-No (*n* = 12)	9.58 ± 1.73	4.67 ± 2.64	−4.92	−6.6	−3.24	< 0.002 ^a^*			
**T1 slope**	MCID-Yes	25.16 ± 3.88	22.13 ± 2.99	−3.04	−3.98	−2.1	< 0.001 ^a^**	−0.87(−4.17 to 2.44)	0.594 ^b^	0.003* ^c^
	MCID- No	30.5 ± 3.73	28.33 ± 4.25	−2.17	−5.38	1.048	0.118 ^a^			
**T1S-CL**	MCID- Yes	15.36 ± 2.82	15.96 ± 2.14	0.61	−0.09	1.31	0.114 ^a^	−2.14(−4.47 to 0.18)	0.039^b*^	< 0.001 ^c^**
	MCID- No	20.92 ± 3.55	23.67 ± 3.75	2.75	0.5	5.002	0.018 ^a^*			

a: Wilcoxon Signed Rank Test, b: Mann-Whitney U test, c: Analysis of Covariance (ANCOVA), * *p* < 0.05: statistically significant, **p < 0.001: statistically highly significant, T1S-CL: T1slope minus C2-C7 lordosis, MCID: Minimal Clinically Important Difference.

**Table 7:** Inter-rater reliability for alignment measurements (ICC, two-way random, absolute agreement).

Parameter	ICC (Single Measures)	95% CI	Significance (*p*)	Interpretation
C2–C7 Lordosis	0.943	0.907–0.965	< 0.001**	Excellent
T1 Slope	0.985	0.975–0.991	< 0.001**	Excellent
T1S-CL	0.961	0.938–0.976	< 0.001**	Excellent

T1S-CL: T1slope minus C2-C7 lordosis, **p < 0.001: statistically highly significant, ICC: Intraclass Correlation coefficient.

### Predictive performance of preoperative radiological parameters

**Fig. 7:**
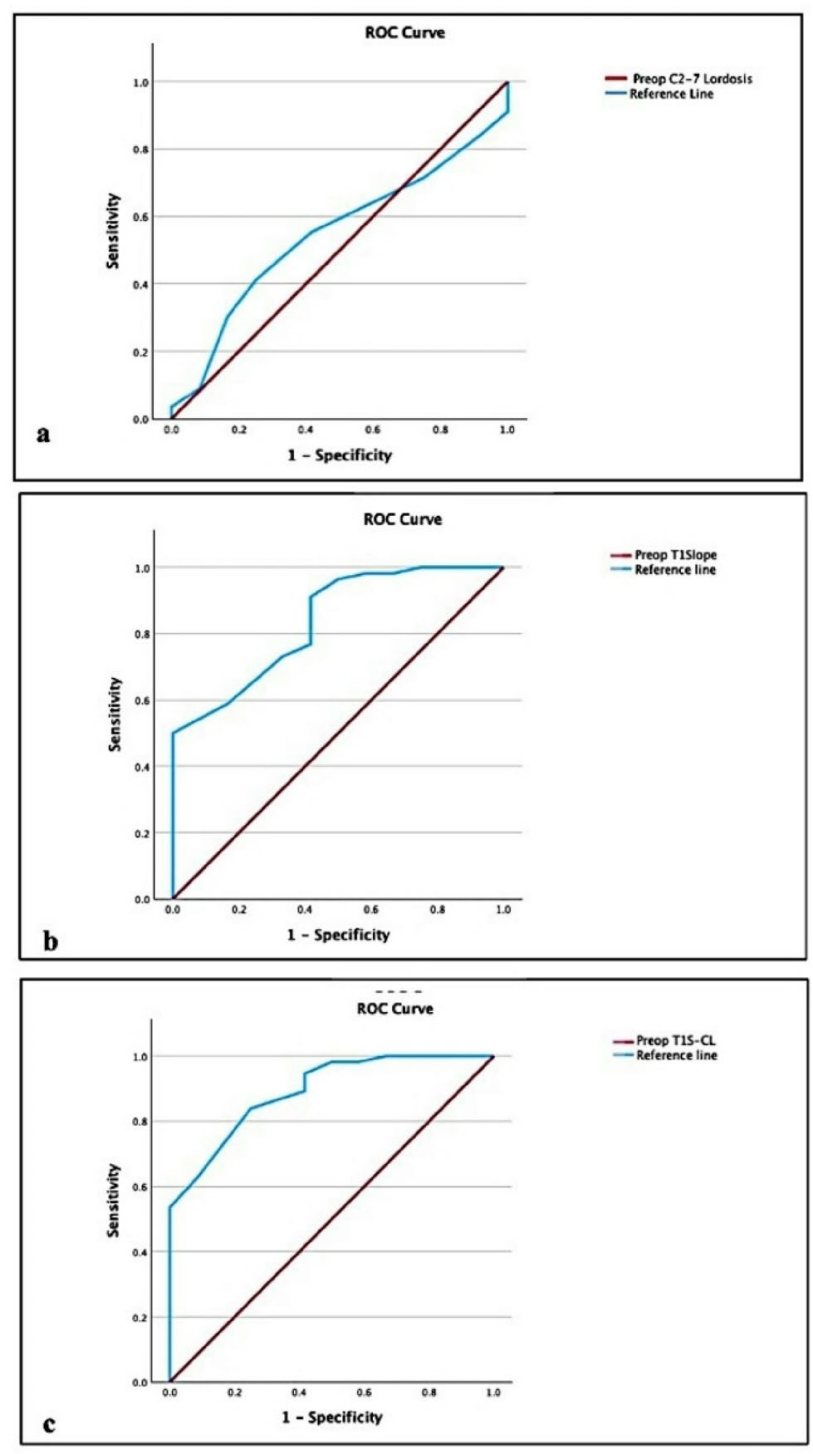
ROC analysis of preoperative radiographic predictors of MCID achievement.

Receiver Operating Characteristic (ROC) analysis was conducted to assess the predictive performance of preoperative radiographic parameters (C2-C7 lordosis, T1S, and T1S-CL). As shown in Table 8, preoperatively, T1S-CL was the strongest predictor for MCID achievement, showing excellent discrimination (AUC 0.893, 95% CI 0.803–0.983) and the largest incremental explanatory power (Nagelkerke R²=0.504). A practical cut-off of 17.5° yielded a balanced profile (sensitivity 83.9%/specificity 75.0%) and a robust per-degree association (OR 0.58, 95% CI 0.44–0.77, *p* < 0.001), with acceptable calibration (Hosmer–Lemeshow *p* = 0.702). T1 slope also discriminated well (AUC 0.837, 95% CI 0.720–0.954), favoring high sensitivity at the 29.5° threshold (91.1%) with moderate specificity (58.3%), and a significant per-degree effect (OR 0.64, 95% CI 0.49–0.83, *p* < 0.001; R²=0.400, Hosmer–Lemeshow *p* = 0.274). In contrast, C2–C7 lordosis performed no better than chance (AUC 0.545, 95% CI 0.384–0.707), and its per-degree effect was non-significant (OR 1.05, 95% CI 0.79–1.39, *p* = 0.751). Collectively, these metrics as demonstrated in Fig. 7, justify selecting T1S-CL as the primary preoperative radiographic predictor for subsequent multivariable modeling.

**Table 8:** Screening performance of preoperative radiographic predictors for MCID achievement.

Predictor	AUC (95% CI)	Youden’s index	Optimal cut-off	Sensitivity/Specificity	OR per 1° (95% CI), *p*	Nagelkerke (*R*²)	Hosmer–Lemeshow (*p*)
**T1S-CL**	0.893 (0.80–0.983.80.983)	0.589	17.5 °	83.9%/75%	0.58 (0.44–0.77), < 0.001**	0.504	0.702
**T1 slope**	0.837 (0.72–0.954)	0.494	29.5°	91.1%/58.3%	0.64 (0.49–0.83), < 0.001**	0.400	0.274
**C2–C7 lordosis**	0.545 (0.384–0.707)	0.161	10.5°	41.1%/75%%	1.05 (0.79–1.39), 0.751	0.002	0.737

T1S-CL: T1slope minus C2-C7 lordosis, AUC: Area Under the Curve, OR: Odds Ratio, **p < 0.001: statistically highly significant, CI: Confidence Interval.

Receiver Operating Characteristic (ROC) analysis comparisons for the predictive performance of preoperative C2-C7 lordosis (a), T1Slope (b), and T1Slope minus cervical lordosis (T1S-CL) (c). T1S-CL shows the highest discrimination. Abbreviations: ROC, receiver operating characteristic; AUC, area under the curve; CI, confidence interval.

#### Model comparison

**Fig. 8:**
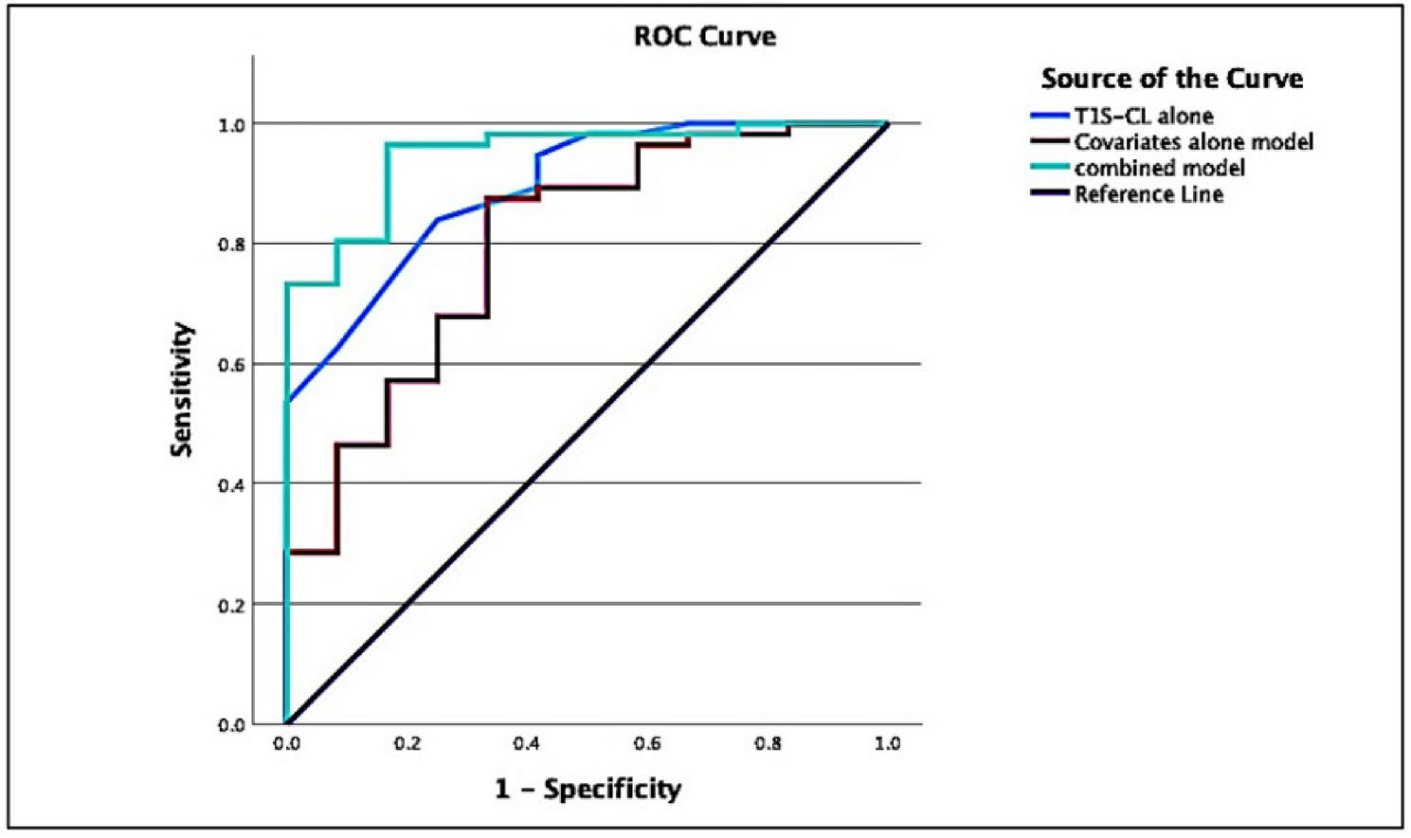
Model comparison: preoperative T1S-CL alone, clinical covariates alone, and combined model. ROC curves comparing discrimination for T1S-CL alone, covariates alone model (age, sex, BMI, symptom duration, number of operated levels, baseline VAS-neck/arm, baseline mJOA), and combined model (T1S-CL + covariates). The combined model achieved the highest AUC. Abbreviations: ROC, receiver operating characteristic; AUC, area under the curve; CI, confidence interval.

As shown in Table 9, ROC analysis and multivariable logistic regression were used to compare the apparent performance of 3 models. Preoperative T1S-CL alone showed excellent discrimination for 2-year MCID achievement (AUC 0.893, 95% CI 0.803–0.983; sensitivity/specificity 83.9%/75.0%; Nagelkerke R² = 0.504; Hosmer–Lemeshow *p* = 0.702). The covariates-only model demonstrated weaker discrimination (AUC 0.799, 95% CI 0.657–0.941; sensitivity/specificity 87.5%/66.7%; Nagelkerke R² = 0.295; Hosmer–Lemeshow *p* = 0.894). The combined model, incorporating T1S-CL and clinical covariates, showed the highest apparent discrimination within the derivation cohort (AUC 0.948, 95% CI 0.890–1.000; sensitivity/specificity 96.4%/83.3%; Nagelkerke R² = 0.657; Hosmer–Lemeshow *p* = 0.733), indicating incremental predictive value beyond clinical factors alone. ROC analysis comparison of the 3 models is shown in Fig. 8.

**Table 9:** Exploratory apparent performance comparison of candidate models in the derivation cohort.

Model	Predictors included	AUC (95% CI)		Youden’s index	Sensitivity/Specificity	Nagelkerke (*R*²)	Hosmer–Lemeshow (*p*)
		AUC	(95% CI)				
**Preoperative T1 slope minus Lordosis alone**	T1S-CL alone	0.893	(0.803–0.983)	0.589	83.9%/75%	0.504	0.702
**Covariates alone**	Covariates alone (age, sex, BMI, symptom duration, operated levels, preop VAS-neck, preop VAS-arm, preop mJOA)	0.799	(0.657–0.941)	0.542	87.5%/66.7%	0.295	0.894
**Combined model**	T1S-CL + covariates	0.948	(0.890–1.00)	0.798	96.4%/83.3%	0.657	0.733

T1S-CL: T1slope minus C2-C7 lordosis, AUC: Area Under the Curve, CI: Confidence Interval.

#### Model internal validation/uncertainty

For the adjusted model, coefficient uncertainty and calibration-related measures were estimated using nonparametric bootstrapping (2,000 resamples; BCa CIs), whereas the reported AUC reflects the apparent discrimination observed in the derivation cohort. As shown in Table 10, the model demonstrated high apparent discrimination (AUC 0.948, 95% CI 0.890–1.000) and good overall accuracy (Brier score 0.0643), with acceptable calibration metrics and adequate fit (Hosmer–Lemeshow *p* = 0.784; Nagelkerke R² = 0.674). After adjustment, larger preoperative T1S-CL and longer symptom duration remained independently associated with lower odds of achieving MCID.

Table 9 presents comparative apparent performance of the candidate models, whereas Table 10 presents the internally validated estimates for the final parsimonious adjusted model in which preoperative T1S-CL and symptom duration remained independently associated with MCID achievement. Given the modest sample size and the limited number of MCID-negative cases, these performance estimates should be interpreted cautiously pending external validation.

**Table 10:** Internal validation and uncertainty metrics for the final multivariable model.

Metric	Estimate	95% CI
**AUC (combined model)**	0.948	0.890–1.00
**Calibration slope**	1.00	0.599–4.68
**Calibration intercept**	0.00	−2.89–1.20
**Brier score**	0.0643	0.0275–0.1071
**Hosmer–Lemeshow p**	0.784	-
**Nagelkerke R²**	0.674	-
		**BCa 95% CI**,** bootstrap p**
**T1S-CL (per 1°)**,** B coefficients**	−0.587	−1.450 to −1.004, < 0.001**
**Symptom duration (per month)**,** B coefficients**	−0.605	−2.118 to − 0.986, 0.011*

T1S-CL: T1slope minus C2-C7 lordosis, AUC: Area Under the Curve, BCa: bias-corrected and accelerated, CI: Confidence Interval, * *p* < 0.05: statistically significant, **p < 0.001: statistically highly significant.

#### Clinically actionable T1S-CL thresholds

**Fig. 9:**
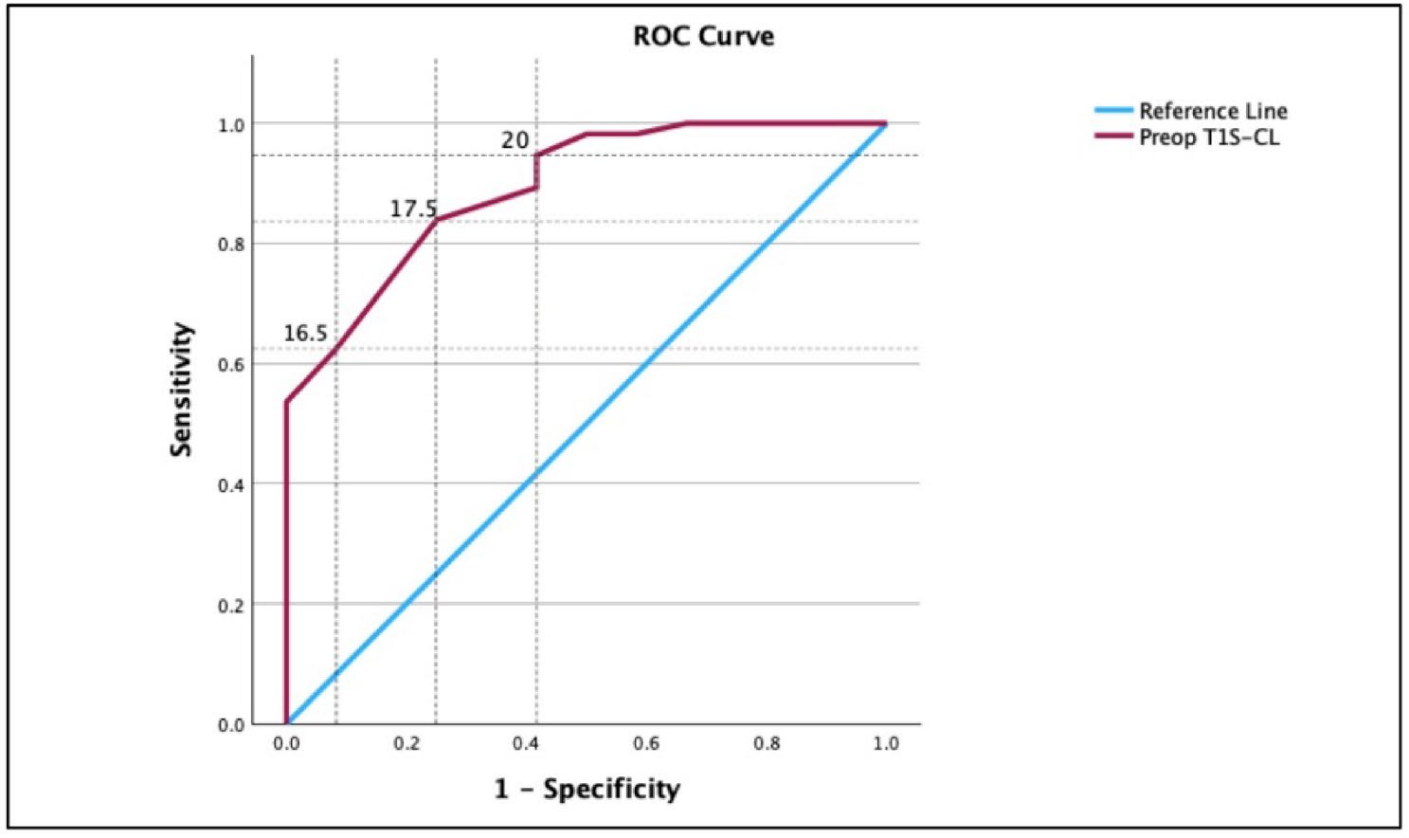
Clinically actionable thresholds for preoperative T1S-CL. ROC curve for T1S-CL annotated at three operating points: ≤16.5° (rule-in; high specificity and PPV), 17.5° (Youden-optimal balance), and > 20° (rule-out; high sensitivity with low LR-). Abbreviations: PPV, positive predictive value; LR-, negative likelihood ratio; T1S-CL, T1 slope minus C2-C7 lordosis.

As shown in Table 11; Fig. 9, three operating points were explored to translate preoperative T1S-CL into preoperative risk stratification. T1S-CL ≤ 16.5° has a high PPV and LR+, suggesting potential utility as an exploratory rule-in threshold for favorable MCID achievement. Conversely, a threshold of > 20° corresponded to high sensitivity and the lowest LR−, suggesting potential utility as an exploratory rule-out threshold for favorable MCID achievement. At 17.5°, T1S-CL provides a balanced operating point (high PPV and good sensitivity with moderate specificity), but it’s less decisive for either confirmation or exclusion. Taken together, these findings suggest a possible two-threshold framework with T1S-CL ≤ 16.5° to rule-in likely MCID and T1S-CL > 20° to rule-out likely MCID, and 16.6–20.0° considered a gray zone in which classification should defer to the multivariable model and clinical context. Because all thresholds were derived from the present cohort, they should be considered hypothesis-generating and not definitive clinical cutoffs, and should be interpreted as an exploratory stratification approach requiring validation in independent cohorts before routine clinical application.

**Table 11:** Clinically actionable preoperative T1S-CL thresholds and performance metrics.

Threshold	Youden’s index	Sensitivity	Specificity	PPV	NPV	LR+	LR-
**T1S-CL ** **16.5°**	0.542	62.5%	91.7%	97.2%	34.4%	7.5	0.41
**T1S-CL 17.5°**	0.589	83.9%	75.0%	94.0%	50.0%	3.36	0.21
**T1S-CL 20°**	*0.530*	*94.6%*	*58.3%*	*91.3%*	70%	2.27	0.093

T1S-CL: T1slope minus C2-C7 lordosis, PPV: Positive Predictive Value, NPV: Negative Predictive Value, LR+: Positive Likelihood Ratio, LR-: Negative Likelihood Ratio.

## Discussion

This study demonstrates that, in elderly patients undergoing standalone laminectomy for degenerative cervical myelopathy (DCM), preoperative T1 slope minus cervical lordosis (T1S-CL) is associated with 2-year functional recovery. T1S-CL outperformed T1 slope and cervical lordosis when evaluated individually and improved model performance when added to clinical covariates. However, because the study was based on a single retrospective cohort of modest size, the predictive model and the proposed thresholds should be interpreted as exploratory rather than definitive. These findings support the relevance of preoperative sagittal alignment in outcome stratification after standalone laminectomy while underscoring the need for external validation before clinical adoption.

### Clinical outcomes

At the 2-year follow-up, our results showed that elderly patients with degenerative cervical myelopathy (DCM) who underwent standalone laminectomy demonstrated significant clinical improvement with more than 82% of patients achieving minimum clinically important difference (MCID) in mJOA scores. Visual analog scale (VAS) scores for neck and arm pain improved from moderate to mild pain levels. These outcomes are consistent with previous studies [21–26]. Nakashima et al.^24^ reported that despite reduced physiological reserve, elderly DCM patients still achieve functionally significant improvement after decompression surgery. A systematic review of 24 studies by Ryken et al.^25^ reported postoperative clinical improvement rates ranging from 42% to 90%. Functional improvements in gait and posture have also been demonstrated^26–28^. Additionally, Kire et al.^29^ documented long-term outcomes in a retrospective series of 110 DCM patients undergoing laminectomy alone, with significant reductions in VAS and Nurick scores and notable mJOA improvements. These findings support laminectomy as a viable intervention for elderly patients, particularly in the absence of preoperative kyphotic deformity.

### Radiological outcomes

Radiologically, our cohort experienced mild but statistically significant changes in sagittal cervical alignment over 2 years. There was a modest reduction in C2–7 lordosis (around 4°) and a slight increase in T1 slope minus cervical lordosis (T1S-CL). This pattern aligns with the mid-term findings reported by Jentzsch et al.^10^, who observed an average of 6° lordosis decrease post-laminectomy. Similarly, Aleixo et al.^30^ reported that only 1 in 57 patients developed mild kyphosis and none exhibited progressive deformity.

Subgroup analysis in our study revealed that patients failing to achieve MCID exhibited less favorable alignment postoperatively, including a greater loss of lordosis and increased T1S-CL. These differences suggest a potential association between sagittal balance and clinical recovery. Sakamoto et al.^31^ similarly reported that a postoperative T1S-CL > 20° was significantly associated with worse Neck Disability Index (NDI) and EuroQol outcomes following laminoplasty. Moreover, Suda et al.^32^ demonstrated that preoperative kyphosis adversely affected neurological recovery due to insufficient posterior cord shift, underscoring the importance of sagittal alignment. Collectively, our findings support the premise that even subclinical sagittal imbalance, as reflected by elevated T1S-CL, may impair outcomes following decompression.

### Predictive performance of T1S-CL

A key aim of our study was to evaluate the prognostic power of preoperative T1S-CL for patient clinical outcomes, in comparison to the individual parameters of T1 slope and C2-C7 lordosis alone. The preoperative T1S-CL emerged as the most robust predictor of postoperative outcome compared to T1 slope or cervical lordosis alone. With an area under the ROC curve (AUC) of 0.89, T1S-CL demonstrated excellent discriminative ability, outperforming T1Slope (AUC = 0.84) and C2–7 lordosis (AUC = 0.55). This supports the hypothesis that T1S-CL represents the relationship between the thoracic inlet angle and required cervical curvature, akin to pelvic incidence–lumbar lordosis (PI–LL) mismatch in the lumbopelvic spine^15,16^. To our knowledge, this study is among the first to directly compare T1S-CL against T1 slope and lordosis head to head in an outcome prediction context and our findings provide evidence validating T1S-CL as a critical alignment parameter in DCM.

Our findings align with a growing body of literature emphasizing the importance of sagittal cervical alignment on surgical outcomes in DCM. Prior work by Staub et al^16^. established that in asymptomatic adults, the normal relationship between T1 slope and cervical lordosis follows the formula CL = T1S – 16.5° ± 2°. The current cervical deformity classification considers a T1S-CL > 20° as mismatch, whereas < 20° is deemed a harmonious or “matched” alignment^15^. This 20° threshold has been assessed in clinical studies, and Rao et al.^17^ reported that DCM patients with preoperative T1S-CL > 20° had a markedly higher incidence of post-laminoplasty kyphotic alignment and significantly worse postoperative NDI and JOA recovery rates. Consistently, our results in a pure laminectomy series show that even among patients lacking gross cervical kyphosis, those with a larger T1S-CL mismatch experienced poorer functional gains, reinforcing that subtle preoperative sagittal imbalance can predispose to suboptimal recovery.

It is worth noting that not all studies have identically quantified the impact of T1S-CL, likely due to differences in patient selection and surgical technique. Sakamoto et al.^31^ focused on laminoplasty patients without severe preoperative kyphosis and found that a T1S-CL > 20° before surgery was not significantly associated with worse outcomes in that specific subgroup. However, they observed that a postoperative T1S-CL mismatch was strongly linked to inferior NDI and EuroQol scores, as 21% of their patients developed a > 20° mismatch after laminoplasty. These findings suggest that maintaining sagittal alignment throughout treatment is critical as patients who may tolerate a moderate baseline mismatch could still fail to achieve MCID if alignment deteriorates after decompression. In our cohort of laminectomy patients, we similarly noted cases of progressive lordosis loss and increasing T1S-CL over 2 years, which coincided with worse clinical improvement.

### Integrating T1S-CL and clinical covariates: multivariable predictive model

In this study, we evaluated the incremental prognostic value of combining T1S-CL with key baseline clinical variables to predict achievement of MCID after standalone laminectomy. At the model-comparison level, the combined model showed higher apparent discrimination than either T1S-CL alone or clinical covariates alone, suggesting that sagittal alignment adds meaningful prognostic information beyond conventional clinical factors. Given that only 12 patients failed to achieve MCID, however, coefficient-level interpretation was intentionally focused on a parsimonious adjusted model to reduce overfitting risk.

Within that adjusted framework, larger preoperative T1S-CL and longer symptom duration remained independently associated with lower odds of achieving MCID. These findings are consistent with prior literature linking sagittal imbalance and delayed intervention to less favorable recovery in DCM. At the same time, the very high apparent AUC observed in the present cohort should be interpreted cautiously, as model performance in a small retrospective derivation sample may be optimistic even when bootstrap resampling is used. Accordingly, our results support T1S-CL as a promising prognostic marker and as a candidate component of a future clinical prediction tool, but not yet as a fully validated instrument for routine decision-making.

These findings expand on prior literature highlighting the prognostic relevance of cervical alignment parameters, such as in studies by Iyer et al.^33^ and Kim et al.^12^which associated T1S-CL mismatch with worse patient-reported outcomes and alignment progression. Broader DCM literature has identified prolonged symptom duration as a risk factor for incomplete recovery^34^^35^,.

The strong impact of symptom duration and cord signal changes on outcome has been demonstrated in other models such as that of Zhang et al.^36^, who combined age, duration, and MRI signal intensity ratio to stratify DCM surgery prognosis, and reported that the presence of ≥ 2 risk factors (age ≥ 63, duration ≥ 9 months, high T2 signal ratio) corresponded to dramatically increased odds of a poor outcome. However, few studies have embedded T1S-CL into a multivariable prognostic framework or tested its incremental value beyond clinical factors. Our model suggests that both biomechanical alignment and disease chronicity are critical for determining surgical benefit.

The multivariate model concept can serve as a personalized risk calculator, guiding whether additional interventions are warranted. This concept parallels the recommendations of Hsu et al.^37^, who recently used a combination of factors (Central canal stenosis, preoperative neck pain and a K-line during flexion) to guide surgical approach selection for laminoplasty patients. They suggested that if certain risk determinants are present (indicating potential for postoperative neck dysfunction), the standard posterior approach should be considered.

Interestingly, within our exclusively elderly cohort (all age ≥ 65 years by design), chronological age was not a significant predictor, likely because of the narrow age range. Age has shown mixed results in literature, with some studies find older age to predict poorer functional gains^36,38,39^, while others^40–42^, including a review by Tetreault et al.^35^ found that age by itself is not a definitive predictor when other factors are controlled, which could be explained as age acting partly as a proxy for frailty or chronicity, which were captured more directly by other variables.

### Clinically actionable T1S-CL thresholds

To facilitate clinical application, we explored ROC-derived operating points for preoperative T1S-CL. A value ≤ 16.5° provided high specificity (91.7%), positive predictive value (97.2%), and LR+ (7.5), making it as possible rule-in threshold for favorable outcomes. Conversely, T1S-CL > 20° yielded high sensitivity (94.6%) and the lowest LR− (0.093), supporting its possible role as a rule-out criterion. These findings suggest that low T1S-CL values may identify patients more likely to benefit from standalone laminectomy, while marked mismatch may identify patients in whom recovery is less predictable. Nevertheless, these thresholds were derived from the same cohort in which model performance was assessed and therefore should be considered exploratory. Rather than definitive cutoffs, they are best viewed as candidate thresholds for future validation studies and as adjuncts to, rather than replacements for, comprehensive clinical judgment. This dual-threshold approach expands on previous work by Wang et al.^18^ and Protopsaltis et al.^15^, who identified associations between T1S-CL and quality of life but did not delineate clear cutoffs.

### Complications

Surgical site infection occurred in 7.4% of patients, with only two cases requiring reoperation for debridement, aligning with published infection rates ranging from 4% to 10% following posterior cervical surgery^43^. C5 palsy, a known complication of posterior decompression, was observed in 4.4% of patients, aligns with the reported incidence of 6.3%^44^ de Dios et al.^45^ reported higher complication rates in the fusion group (24 per 100 operations) than in the laminectomy alone group (11 per 100 operations). They also reported more superficial surgical site infections and a higher reoperation rate in the fusion group. Non-neurological complications, including postoperative chest infection (5.9%) and deep vein thrombosis (1.5%), were similarly within the expected limits for an elderly surgical population and did not differ significantly between outcome subgroups. These findings suggest that standalone laminectomy can be performed with acceptable morbidity rates in well-selected older adults.

### Clinical implications and future directions

This study highlights T1 slope minus cervical lordosis (T1S-CL) as a simple radiographic parameter that may improve preoperative risk stratification in elderly patients undergoing laminectomy for degenerative cervical myelopathy (DCM). Even among patients without overt kyphosis, those with a preoperative T1S-CL exceeding 20° experienced less clinical improvement, suggesting that subtle sagittal imbalance may compromise outcomes. Incorporating T1S-CL into preoperative assessment may refine preoperative counseling and support individualized surgical planning.

However, the present findings should not be interpreted as sufficient to mandate specific surgical alternatives solely on the basis of T1S-CL. The multivariable model and the proposed thresholds were internally derived within a single retrospective cohort and now require external validation in independent populations. Future prospective, multi-center studies are warranted to externally validate these findings and assess whether addressing mismatch through alignment-correcting strategies improves long-term outcomes. Such efforts will refine surgical algorithms and support personalized spine care in the aging population with DCM.

### Limitations and strengths

This study had some limitations. First, as a retrospective analysis, it is subject to selection bias for which we followed strict eligibility criteria for the inclusion of consecutive patients. Second, unmeasured confounders, including frailty, muscular integrity, or global spinal balance, may have influenced the outcomes. Third, follow-up was limited to 2 years with long-term functional outcomes remain uncertain. Fourth, although the study cohort included 68 patients, only 12 patients did not achieve MCID, thus, the final adjusted model had an events-per-variable ratio of approximately 6, which is below the conventional rule of thumb of 10 and increases the risk of overfitting despite bootstrap-based internal validation. Finally, the predictive model and the proposed T1S-CL thresholds were derived and tested within the same cohort; therefore, their generalizability and clinical applicability remain unproven pending external validation.

Despite these limitations, the study also has important strengths. It addresses a clinically relevant and relatively underexplored question in a homogeneous cohort of elderly patients treated with standalone laminectomy. It directly compares T1S-CL with T1 slope and cervical lordosis as prognostic radiographic measures and demonstrates the incremental value of incorporating sagittal alignment into outcome prediction. In addition, the study used a structured modeling framework with prespecified covariates, ROC-based assessment of discrimination, and bootstrap resampling to characterize coefficient uncertainty and calibration-related measures. These strengths support the view that T1S-CL is a promising prognostic marker in this setting, while also emphasizing that further validation is required before routine clinical implementation.

## Conclusion

Standalone laminectomy yields meaningful clinical improvement in elderly patients with degenerative cervical myelopathy. In this exploratory cohort, preoperative T1 slope minus cervical lordosis (T1S-CL) was strongly associated with 2-year functional recovery and added prognostic information beyond baseline clinical variables. Internally derived thresholds suggested potential utility for preoperative risk stratification, with lower mismatch associated with more favorable outcomes and marked mismatch associated with less predictable recovery. However, the predictive model and proposed thresholds were derived from a single retrospective cohort and require external validation before routine clinical application.

## Supplementary Information

Below is the link to the electronic supplementary material.

Supplementary Material 1

## Data Availability

The datasets generated during and/or analysed during the current study are available in the Harvard Dataverse repository, [https://doi.org/10.7910/DVN/KU7AUD].

## Abbreviations

DCM: degenerative cervical myelopathy
VAS: Visual Analog Score
MRI: Magnetic Resonance Imaging
CT: Computed Tomography
DM: diabetes mellitus
HTN: hypertension
IHD: ischemic heart disease
SD: standard deviation
ACDF: anterior cervical discectomy and fusion
mJOA: modified Japanese Orthopaedic Association score
NDI: Neck Disability Index
MCID: minimal clinically important difference
T1S: T1 Slope
T1S-CL: T1 Slope minus C2–C7 lordosis
OPLL: ossification of the posterior longitudinal ligament
ANCOVA: Analysis of Covariance
ROC: Receiver Operating Characteristic analysis
BCa: bias‑corrected and accelerated
PPV: positive predictive value
NPV: negative predictive value
LR+: Positive Likelihood Ratio
LR-: Negative Likelihood Ratio
