# Supplementary material for: Predictive value of preoperative T1 slope minus cervical lordosis for clinical outcomes after standalone laminectomy in elderly degenerative cervical myelopathy
Source: Sci Rep. 2026 Apr 13;16:12356. doi: 10.1038/s41598-026-46868-1 (PMC13079728; doi:10.1038/s41598-026-46868-1)
Supplement: Supplementary file 1 — Supplementary Material 1 [file 41598_2026_46868_MOESM1_ESM.pdf]

Supplementary Material

1- ROC analysis syntax

1.1. comparison between C2-C7cervical lordosis (CL), T1Slope (T1S), and T1 Slope minus C2-C7 lordosis (T1S-CL):

```
ROC ANALYSIS preop_C2_C7_Lordosis BY MCID_Achieved (1)
  /MISSING USERMISSING=EXCLUDE
  /CRITERIA CUTOFF=INCLUDE TESTPOS=LARGE DISTRIBUTION=FREE CI=95
  /DESIGN PAIR=FALSE
  /PLOT CURVE=ROC(REFERENCE) MODELQUALITY=FALSE
  /PRINT SE=TRUE COORDINATES=ROC(YOUDEN) CLASSIFIER=FALSE.

ROC ANALYSIS preop_T1_slope BY MCID_Achieved (1)
  /MISSING USERMISSING=EXCLUDE
  /CRITERIA CUTOFF=INCLUDE TESTPOS=SMALL DISTRIBUTION=FREE CI=95
  /DESIGN PAIR=FALSE
  /PLOT CURVE=ROC(REFERENCE) MODELQUALITY=FALSE
  /PRINT SE=TRUE COORDINATES=ROC(YOUDEN) CLASSIFIER=FALSE.

ROC ANALYSIS preop_T1SlopeminusC27_lordosis BY MCID_Achieved (1)
  /MISSING USERMISSING=EXCLUDE
  /CRITERIA CUTOFF=INCLUDE TESTPOS=SMALL DISTRIBUTION=FREE CI=95
  /DESIGN PAIR=FALSE
  /PLOT CURVE=ROC(REFERENCE) MODELQUALITY=FALSE
  /PRINT SE=TRUE COORDINATES=ROC(YOUDEN) CLASSIFIER=FALSE.
```

1.2. Model comparison: T1S-CL alone, covariates alone model, and combined model:

```
ROC ANALYSIS PRE_5_combined_model PRE_4_covariates_alone_model
PRE_3_T1S_Minus_CL BY MCID_Achieved
   (1)
  /MISSING USERMISSING=EXCLUDE
  /CRITERIA CUTOFF=INCLUDE TESTPOS=LARGE DISTRIBUTION=FREE CI=95
  /DESIGN PAIR=FALSE
  /PLOT CURVE=ROC(REFERENCE) MODELQUALITY=FALSE
  /PRINT SE=TRUE COORDINATES=ROC(YOUDEN) CLASSIFIER=FALSE.
```

2- Bootstrap

Syntax:

```
BOOTSTRAP
  /SAMPLING METHOD=SIMPLE
  /VARIABLES TARGET=MCID_Achieved INPUT=Age sex pre_vas_neck pre_vas_arm
Preop_mJOA levels_operated
    BMI Symptom_Duration_Months preop_T1SlopeminusC27_lordosis
  /CRITERIA CILEVEL=95 CITYPE=BCA  NSAMPLES=2000
  /MISSING USERMISSING=EXCLUDE.
LOGISTIC REGRESSION VARIABLES MCID_Achieved
  /METHOD=ENTER Age sex pre_vas_neck pre_vas_arm Preop_mJOA levels_operated BMI
    Symptom_Duration_Months preop_T1SlopeminusC27_lordosis
  /CONTRAST (sex)=Indicator(1)
  /PRINT=GOODFIT CI(95)
                        /CRITERIA=PIN(0.05) POUT(0.10) ITERATE(20) CUT(0.5).
```

Output:

Table 1. Variables in the equation

	**B**	**Wald**	**Significance**	**Exp(B)**	**95% C.I for EXP(B)**	
					Lower	Upper
**Age**	.058	.113	.737	1.060	.755	1.489
**sex (1)**	.033	.001	.978	1.033	.105	10.153
**Preop. VAS Neck**	.206	.270	.603	1.228	.566	2.667
**Preop. VAS Arm**	.095	.065	.799	1.100	.529	2.287
**Preop. mJOA**	-.127	.160	.689	.881	.473	1.641
**Levels operated**	-.249	.064	.801	.779	.112	5.419
**BMI**	-.054	.039	.843	.947	.554	1.620
**Symptom Duration, months**	-.605	3.466	.063	.546	.289	1.032
**Preop T1Slope minus C2-C7 lordosis**	-.587	9.979	.002*	.556	.386	.800

, * p< 0.05: statistically significant

Table 2. Bootstrap of variables in the equation

	B	Bootstrap		
		Significance (2-tailed)	BCa 95% Confidence Interval	
			Lower	Upper
**Age**	.058	.466	-25.029	24.015
**sex (1)**	.033	.662	-184.276	138.978
**Preop. VAS Neck**	.206	.356	-92.853	133.419
**Preop. VAS Arm**	.095	.495	-56.837	86.982
**Preop. mJOA**	-.127	.412	-50.264	40.273
**Levels operated**	-.249	.520	-89.111	6.476
**BMI**	-.054	.479	-28.043	18.347
**Symptom Duration, months**	-.605	.011*	-2.118	-.986
**Preop T1Slope minus C2-C7 lordosis**	-.587	<.001**	-1.450	-1.004

, * p< 0.05: statistically significant, **p< 0.001: statistically highly significance.
